# Analysis of tennis champions’ career: how did top-ranked players perform the previous years?

**DOI:** 10.1186/2193-1801-3-504

**Published:** 2014-09-07

**Authors:** Javier Maquirriain

**Affiliations:** Argentine Tennis Association, CONICET, Buenos Aires, Argentine

**Keywords:** Ranking, Sport, Professional

## Abstract

Professional tennis is a highly competitive sport ranked through an objective, merit-based, mathematical system. The objective of this study was to determine and analyze how top 1°-ranked professional tennis players perform in the previous three years. Data of ranking position of all top 1°-ranked female and male players in the professional era and their performance in Grand Slams tournaments were collected from tennis stakeholders’ websites and analyzed.

Top 1° male players’ ranking position averaged 2.17 ± 1.92 (CI 95%:1.56/2.78), 7.02 ± 18.073, and 10.73 ± 29.31, at 1, 2, and 3 previous years, respectively. Top 1° female players’ ranking position averaged 2.21 ± 1.59 (CI 95%:1.61/2.71), 4.78 ± 4.09, and 14.97 ± 26.75, at 1, 2, and 3 previous years, respectively. All top 1° male and female players were ranked within the 1°-10° and 1°-6° positions the previous year, respectively; the majority of tennis champions won at least one Grand Slam tournament during the year before reaching the top 1° ranking position (females = 69%; males = 65%), and during the same season (females = 82%; males = 92%). Female and male top 1°-ranked player maintained that position in the following year in 48.7% and 52.5% of cases, respectively.

In conclusion, tennis players need to perform at high level during at least three years prior to reach the top 1° position in the professional tennis ranking. Both, male and female champions, showed similar patterns of performance in their professional career.

## Background

The majority of professional sports have incorporated statistics to evaluate or forecast matches outcomes. The sport of tennis has lagged behind most its peers in collecting statistics data. However, there is a growing interest in this topic and several studies on tennis rankings and career development have been recently published (Bane et al. 2014; Reid et al. 2014; Reid & Morris 2011; Brouwers et al. 2012; Radicchi 2011). The career of professional tennis players are measured by the achievement of ranking milestone (Reid et al. 2014). These authors consider that professional tennis rankings provide valuable but underutilized information (Reid et al. 2014).

The *International Tennis Federation* (ITF), the *Women Tennis Association* (WTA), and the *Association of Tennis Professionals* (ATP) are the principal organizing bodies of women and men professional tennis, respectively. The ATP was formed in 1972 by Donald Dell, Jack Kramer, and Cliff Drysdale to protect the interests of male professional tennis players. One of the initial acts of the organization was the establishment of a computer ranking system that provided fair analysis of a player’s performance as well as an objective means to determine the entries into tournaments. It was created by Jack Kramer, started the following year (23 August, 1973), and has continued through today as the official ranking system in men’s professional tennis (Association of Tennis Professional web site, 2014). The WTA was founded in 1973 by Billie Jean King and colleagues. Jerry Diamond is considered the creator the women’s point system for ranking players which started in 1975 (Women Tennis Association web site, 2014).

The ITF supervise the four Grand Slam (GS) tournaments, also called “majors”, which are the most important annual tennis events and offer the most ranking points and prize money. The Grand Slam itinerary consists of the Australian Open, the French Open (Roland Garros), Wimbledon, and the US Open.

The objective of this descriptive study was to determine and analyze how top-ranked professional tennis players, male and female, performed the previous three years.

## Methods

The protocol of this observational study was approved by the Ethics Committee of the Nixus Foundation (Permit Number: 2013–07). Informed consent was not required but subjects’ ranking positions were de-identified prior to analysis.

Both professional tennis rankings (ATP and WTA) are objective, merit-based, mathematical method of ranking tennis players. These international organizations publish weekly lists, a 52-week rolling computer ranking points based on tournament category. The player with the most points by season’s end is the World Number 1 of the year.

Data of ranking position of all top 1°-ranked female and male players in the professional era and their performance in Grand Slams tournaments were collected from tennis stakeholders’ websites available in the public domain and analyzed. (Association of Tennis Professional web site, 2014; Women Tennis Association web site, 2014).

Statistical analysis was performed using Statistica™ software (*StatSoft Inc, Tulsa, Oklahoma*). Ordinal variables were analyzed through the Mann–Whitney test; t-test was used for other comparisons; *p* < 0.05 was considered significant.

## Results

### Male circuit

There were 16 different players who reached the top-1° position of the ATP ranking at the end of the year during the professional era (1973–2013).

The mean ranking position the year before reaching the top-1° place was 2.17 ± 1.92 (range 1–10; CI 95%: 1.56-2.78; CI 99%: 1.35-2.99). The first player who reached the top position was not used for analysis because official data regarding his performance during previous years were not available. Data regarding their position in the previous three years are shown in Table 1.

**Table 1:** **Descriptive statistics of top 1° male players’ performance in the ATP year-end ranking**

Previous year	n	Average	SD	Range	CI 95%
1	40	2.17	1.92	1-10	1.56/2.78
2	39	7.02	18.07	1-110	1.16/12.88
3	38	10.73	29.31	1-156	1.10/20.37

Data included their ranking position at three previous years.

All top 1° players (100%) ranked within the 1°-10° positions the previous year before reaching the peak place. In 52.50% of cases (21/40), the World Number 1 of the year was the same player as the previous season.

Ninety-seven percent of top 1° players (38/39) were ranked within the 1°-25° positions two years before reaching the peak position. Ninety-four percent of top-1 players (36/38) were ranked within the 1°-25° positions three years before reaching the peak position.

The analysis of top 1° players performance in GS tournaments showed that 65.85% (27/41) had won at least one GS tournament the previous year (fourteen players won one GS tournament; eleven players won two GS tournaments; two players won three GS tournaments). The previous year, top-1° players won Wimbledon (n = 15), US Open (n = 14), Roland Garros (n = 7), and Australian Open (n = 6). The difference between GS winners and GS non-winners in the previous years was not statistically significant (*p* = 0.24; chi-square test).

Ninety-two percent of top 1° players (38/41) had won at least one GS tournament during the year when they reached that position in the final ranking. Fifteen players won one Grand Slam tournament; fifteen won two Grand Slam tournaments; and seven players won three Grand Slam tournaments. During that season, players won US Open (n = 22), Wimbledon (n = 21), US Open (n = 22), Roland Garros (n = 12) and Australian Open (n = 11) Table 2.

**Table 2:** **Descriptive statistics of performance in Grand Slam tournaments of male players who reached the 1** ^**st**^ **position of ATP ranking**

Season	Winners	%	GS tournaments	Average
Same year	38/41	92.68	67/38	1.76
1° previous year	27/41	65.85	42/27	1.55
2° previous year	22/41	53.65	36/22	1.63
3° previous year	23/41	56.09	34/23	1.47

There were 16 different male players who reached the top-1° ranking position at the end of the calendar year in the professional era. At the year when they reached that position for the first time, all of them (16/16) had won at least one Grand Slam tournament (average 1.81). Their performance in Grand Slam tournaments the previous years included are summarized in Table 3.

**Table 3:** **Descriptive statistics of performance in Grand Slam tournaments of male players who reached the 1** ^**st**^ **position of ATP ranking for the first time**

Season	Winners	%	GS tournaments	Average
Same year	16/16	100.00	29/16	1.81
1° previous year	7/16	43.75	8/16	0.50
2° previous year	4/16	25.00	4/16	0.25
3° previous year	7/16	43.75	7/16	0.43

### Female circuit

There were 11 different players who reached the top-1° position of the WTA ranking at the end of the year during the professional era. The mean ranking position the year before reaching the top-1° place was 2.21 ± 1.59 (range 1–6; CI 95%: 1.68-2.73; CI 99%: 1.50-2.91). Again, the first player who reached the top position was not used for analysis because official data regarding her performance during previous years were not available. Data regarding their position in the previous three years are shown in Table 4.

**Table 4:** **Descriptive statistics of top 1° female players’ performance in the WTA year-end ranking**

Previous year	N	Average	SD	Range	CI 95%
1	38	2.21	1.59	1-6	1.68-2.73
2	37	4.78	4.09	1-16	3.42-6.14
3	36	14.97	26.75	1-95	5.92-24.02

Data included their ranking position at three previous years.

All top-1° female players (100%) ranked within the 1°-6° positions the previous year before reaching the peak place. In 48.71% of cases (19/39), the World Number 1 of the year was the same player as the previous season.

Ninety-seven percent of top 1° players (36/37) were ranked within the 1°-12° positions two years before reaching the peak position. Eighty-six percent of top 1° players (31/36) were ranked within the 1°-22° positions three years before reaching the peak position.

Sixty-four percent of top-1° players (25/39) had won at least one GS tournament the previous year (eleven players won one GS tournament; nine players won two GS tournaments; four players won three GS tournaments, and 1 player won four GS). In the previous year, top 1° players won Wimbledon (n = 13), Roland Garros (n = 13), and Australian Open (n = 10), and the US Open (n = 9). The difference between GS winners and GS non-winners in the previous year was not statistically significant (*p* = 0.22; chi-square test).

Eighty-two percent of top 1° female players (32/39) had won at least one Grand Slam Tournament during the year when they reached that position in the final ranking. Thirteen players won one Grand Slam tournament; eight won two Grand Slam tournaments; ten players won three Grand Slam tournaments; and one player won four Grand Slam tournaments. During that season, players won the US Open (n = 19), Wimbledon (n = 17), Roland Garros (n = 15), and the Australian Open (n = 12). Performance pattern of female tennis champions in GS tournaments are summarized in Table 5.

**Table 5:** **Descriptive statistics of performance in Grand Slam tournaments of female players who reached the 1** ^**st**^ **position of ATP ranking**

Season	Winners	%	GS tournaments	Average
Same year	32/39	82.05	64/32	2.00
1° previous year	27/39	69.23	47/27	1.74
2° previous year	23/39	58.97	39/23	1.69
3° previous year	18/39	46.15	29/18	1.61

There were 11 different female players who reached the top 1° ranking position at the end of the calendar year in the professional era. At the year when they reached that position for the first time in their career, eighty-one percent of them (9/11) had won at least one Grand Slam tournament. Their performance in Grand Slam tournaments the previous years included are summarized in Table 6.

**Table 6:** **Descriptive statistics of performance in Grand Slam tournaments of female players who reached the 1** ^**st**^ **position of WTA ranking for the first time**

Season	Winners	%	GS tournaments	Average
Same year	9/11	81.81	17/9	1.88
1° previous year	2/11	18.18	3/2	1.50
2° previous year	0/11	0.00	0/0	0.00
3° previous year	1/11	9.09	1/1	1.00

### Male vs. female comparison

There were no significant differences between male and female performance pattern at previous years before reaching the top 1° ranking position. Data are summarized in Table 7.

**Table 7:** **Gender comparison of players’ performance before reaching the top 1° ranking position**

	Male	Female	*p*
**Previous ranking at 1 year**	2.17 ± 1.92	2.21 ± 1.59	0.70
**Previous ranking at 2 years**	7.02 ± 18.07	4.78 ± 4.09	0.44
**Previous ranking at 3 years**	10.73 ± 29.31	14.97 ± 26.75	0.83
**All top-1° ranked at 1 year**	1°- 10°	1°- 6°	
**Same player top-1° as past year**	52.50%	48.71%	0.73
**Grand Slam winner the year before**	65.85%	69.23%	0.86
**Grand Slam winner the same year**	92.68%	82.05%	0.15
**Grand Slam winner the same year in # 1 players for first time**	100.00%	81.81	0.07

## Discussion

Findings of this study indicated that male and female tennis champions need to perform at high level the previous years before reaching the top 1° position. In the overall professional era (40 consecutive years, 1974–2013 for men; and 38 consecutive years, 1976–2013 for women) the average of their ranking position the year before reaching the top of the list was “2°”, with really close data dispersion. Furthermore, all of them (100%) were ranked within the top-6° (females) and the top-10° positions (males) the previous year. Confidence intervals of descriptive statistics (Cummings and Koepsell 2010) showed that there is 95% of chance that the next top 1° World player (male and female) will be one player ranked 1°, 2°, or 3° at the present year.

Two years before reaching the top-1° ranking position, male players averaged the 7° place in the ATP year-end ranking, and female players averaged 5° in the WTA ranking system. The vast majority (94%) of male players was ranked within the 25° position at that time, and there was 95% of chance that the top 1° player in two years will be ranked within the 1-13° positions at the present season. In the female circuit, 97% of players were ranked within the 12° position two years before reaching the top position, and there is 95% of possibility that the top 1° player in two years will be ranked within 3°-6° positions at the present year.

Three years before climbing to the top-1° ranking position, players average the 11° place in the ATP year-end ranking. Most of them (94%) were ranked within the 25° position at that time. Moreover, there is a 95% of chance that the top 1° player in three years will be ranked within 1°-20° positions at the present season.

The four GS tournaments offer the most ranking points in the professional tennis circuit; therefore, they have a clear influence in the final ranking constitution every year. Undoubtedly, winning a major is considered a cornerstone in the tennis player’s career. The majority of tennis champions won at least one GS tournament during the year before reaching the top 1° ranking position (females = 69%; males = 65%), and during the same season (females = 82%; males = 92%). Moreover, in the male circuit it seems necessary to win two majors in the same season to become top 1° for the first time.

High level of performance consistency of top 10°-ranked players has been previously reported (Maquirriain and Cerúndolo 2005; Rohm et al. 2004). It seems to be extremely necessary to perform at very high level during at least three years prior to become top 1° in male professional ranking. Potential explanation for this issue may be the high competitiveness of the professional circuit, the seeding procedure for drawing the tournaments’ matches, and the intrinsic rolling computer system of the ranking, which maintains points during 52 weeks.

Both male and female professional tennis champions showed similar pattern regarding ranking evolution and other performance data. Top 1° players did not show gender differences in their ranking position in the previous years and overall Grand Slam tournaments. The main difference was found in the rate of majors’ winners during the season in which the player reached the top position for the first time. All male champions won a GS tournament that year, whereas some female players (18%) were able to become a new top 1° without winning a major event.

Limitations of the current study include its descriptive and retrospective design. Further researches are needed to really predict future positions in tennis rankings.

## Conclusions

According to the findings of this study, it seems to be extremely necessary to perform at very high level during at least three years prior to become top 1° ranked player in female and male professional tennis circuits. There is 95% probability that the next top 1° ranked player will be one ranked within the 1°-3° positions at the present year, and the top 1° ranked player has approximately 50% of chances to maintain that position the next year. The majority of tennis champions won a Grand Slam tournament during the year before reaching the top 1° ranking position and during the same season. Both, male and female tennis players, showed similar patterns of performance before reaching the top ranking positions.
